# Is it feasible to select fetuses for prenatal WES based on the prenatal phenotype?

**DOI:** 10.1002/pd.5522

**Published:** 2019-09-10

**Authors:** Karin Diderich, Marieke Joosten, Lutgarde Govaerts, Diane Van Opstal, Attie Go, Maarten Knapen, Robert‐Jan Galjaard, Lies Hoefsloot, Malgorzata Srebniak

**Affiliations:** ^1^ Department of Clinical Genetics Erasmus Medical Center Rotterdam The Netherlands; ^2^ Department of Obstetrics and Fetal Medicine Erasmus Medical Center Rotterdam The Netherlands

Recently published studies have shown the feasibility of whole exome sequencing (WES) in prenatal diagnosis.^1^, ^2^ As WES is not yet routinely implemented in all cases of fetal anomalies, discussions arise on which fetuses should be referred for broad genetic testing. The diagnostic yield is highest in fetal cases with multiple congenital anomalies. Therefore, recently Lord and et al^1^ suggested that WES is best performed by targeting those groups, in which it is most likely to be diagnostic, allowing a high diagnostic yield. We do agree that these cases have a clear indication for WES, which will lead to a diagnosis in 15.4%, as shown by Lord et al (2019). This is important knowledge for the parents and relevant information for assessment of the recurrence risk. However, in many of such cases, a prenatal genetic diagnosis does not add significantly to decisions on pregnancy management. Eg, in case of a fetus with short extremities, a narrow thorax and polydactyly, the molecular diagnosis Ellis Van Creveld syndrome does neither change the fetal prognosis, nor perinatal management of the pregnancy. This is also supported by the paper of Lord et al, since 20/52 (38.5%) diagnoses were made in pregnancies that were terminated based solely on the ultrasound abnormalities. We think that the detection of genetic variants as a syndromic cause of fetal anomalies may be especially valuable in prenatal cases with a less severe phenotype or cases with an apparently isolated anomaly, in which a molecular diagnosis has a larger impact on decision‐making during pregnancy or neonatal management. In such cases, parents are facing difficult decisions based on an incomplete phenotype. It is well known that many syndromes have postnatal features that cannot be detected prenatally on routine or expert ultrasound scans, eg, intellectual disability or hypotonia. An example is the case of Lord et al with an atrioventricular canal defect, where a pathogenic variant in the *ANKRD11* gene was detected (KBG syndrome). An intellectual disability in addition to a structural cardiac defect can significantly influence the prospective parents' decision on continuing or terminating the pregnancy. Petrovski et al 2 found a variant in the *L1CAM* gene in a male fetus with agenesis of the corpus callosum. In our practice, we have diagnosed several severe syndromic disorders in fetuses presenting with milder or isolated anomalies, eg, Cornelia de Lange syndrome (NIPBL) in a fetus with mild intra uterine growth restriction and hypospadias and Warsaw Breakage syndrome (*DDX11*) in a fetus with apparently isolated intra uterine growth restriction on a second trimester fetal anomaly scan. Therefore, based on such examples from the literature as well as from our clinical practice, we question whether is it feasible to select fetuses with a high risk for a syndromic disorder based on the severity of the prenatal phenotype and we suggest to offer WES in all cases with fetal anomalies.

We propose to offer prenatal “top‐priority” WES to pregnant couples (regardless of the severity of the fetal anomalies) who are in doubt about decisions on the course of their pregnancy and/or probably would only consider termination of pregnancy when a severe underlying genetic condition is identified. “Priority” WES should be offered to prospective parents who continue the pregnancy regardless of the genetic anomaly, but in which cases, a diagnosis can potentially influence perinatal/neonatal management. And finally, “routine/postpartum” WES could be offered in all cases with fetal anomalies who decide to terminate the pregnancy, in which a genetic diagnosis is mainly valuable to assess the recurrence risk and the diagnostic protocol in future pregnancies.

As advised by the guidelines of the ISPD 2018,^3^ we still offer WES in selected prenatal cases in the setting of a multidisciplinary team. However, we anticipate that, similar to microarray, prenatal WES soon will be routinely offered in all cases with ultrasound anomalies that undergo invasive sampling. Pretest and especially posttest counseling are of vital importance as it should be noted that where a normal result of WES in case of a “mild” prenatal phenotype is reassuring for prospective parents, one can never exclude the presence of an underlying syndrome. After all, not all pathogenic variants can be detected by WES and some of the variants cannot be classified as (likely) pathogenic in the absence of the postnatal phenotype.
